# Maternal Immunization: Nature Meets Nurture

**DOI:** 10.3389/fmicb.2020.01499

**Published:** 2020-07-24

**Authors:** Anja Saso, Beate Kampmann

**Affiliations:** ^1^Faculty of Infectious and Tropical Diseases, London School of Hygiene & Tropical Medicine, London, United Kingdom; ^2^Vaccines and Immunity Theme, MRC Unit The Gambia at LSHTM, Banjul, Gambia

**Keywords:** maternal immunization, vaccine, neonate, infant immunity, placenta, antibody, breast milk

## Abstract

Vaccinating women in pregnancy (i.e., maternal immunization) has emerged as a promising tool to tackle infant morbidity and mortality worldwide. This approach nurtures a ‘gift of nature,’ whereby antibody is transferred from mother to fetus transplacentally during pregnancy, or postnatally in breast milk, thereby providing passive, antigen-specific protection against infections in the first few months of life, a period of increased immune vulnerability for the infant. In this review, we briefly summarize the rationale for maternal immunization programs and the landscape of vaccines currently in use or in the pipeline. We then direct the focus to the underlying biological phenomena, including the main mechanisms by which maternally derived antibody is transferred efficiently to the infant, at the placental interface or in breast milk; important research models and methodological approaches to interrogate these processes, particularly in the context of recent advances in systems vaccinology; the potential biological and clinical impact of high maternal antibody titres on neonatal ontogeny and subsequent infant vaccine responses; and key vaccine- and host-related factors influencing the maternal-infant dyad across different environments. Finally, we outline important gaps in knowledge and suggest future avenues of research on this topic, proposing potential strategies to ensure optimal testing, delivery and implementation of maternal vaccination programs worldwide.

## Introduction

To improve maternal and neonatal health remains a focus of international investment in global health, given that Millenium Goals 4 and 5 were not achieved (Victora et al., 2016). Despite the wider roll-out and availability of vaccines through the Expanded Program of Immunization (EPI) and their significant contribution to the reduction in under-5 morbidity and mortality, there remains a large gap in protection from infectious diseases in newborns: 40% of all mortality in children under the age of 5 now falls into the neonatal period, a third is attributable to potentially preventable infections. This has significant impact on economic development and welfare of the populations affected, primarily in low- and middle-income countries (LMICs) (Liu et al., 2016).

## Can We Achieve Even More With Vaccines?

There is increasing momentum to develop and implement vaccination of women during pregnancy (also called maternal immunization) to prevent specific infections of particular relevance to pregnancy and the newborn (Zaman et al., 2008; Lindsey et al., 2012; Saso and Kampmann, 2016; Switzer et al., 2019). This approach nurtures a ‘gift of nature,’ whereby antibody is transferred from mother to fetus during pregnancy, via the placenta or postnatally in breast milk, in order to provide passive protection against pathogens in the first few months of life. Maternal immunization, therefore, enhances this passive protection, targeting specific antigens.

Two broad aims can be achieved: (1) Pregnant women can be protected against a number of infectious diseases with more severe outcomes in pregnancy (e.g., influenza) and (2) their newborn babies are protected by high titres of antigen-specific IgG antibody against diseases of particular importance in the newborn [e.g., tetanus, pertussis, Group B streptococcus (GBS), Respiratory Syncytial Virus (RSV)] due to their associated susceptibility (e.g., GBS, tetanus) or severity (e.g., pertussis, RSV) and until they develop protective antibody after receiving their own vaccines or following natural exposure.

Depending on geographical location, maternal immunization is already routinely recommended to prevent tetanus, pertussis and influenza with proven safety, immunogenicity and efficacy, but uptake and coverage vary widely (Lindsey et al., 2012; WHO: Global Advisory Committee on Vaccine Safety, 2014; Steedman et al., 2016; Gkentzi et al., 2017; Marchant et al., 2017; Munoz and Jamieson, 2019; Wales et al., 2020). New vaccines for explicit use in pregnancy are likely to become available in the next few years. However, a number of biological and implementation challenges remain to be addressed to harness the full potential of this intervention. In this review, we summarize key knowledge and identify important gaps in our understanding of vaccination in pregnancy and its impact on the neonatal immune system. We deliberately focus on biological opportunities and challenges, and we will briefly address the nevertheless considerable challenges to implementation of this promising intervention.

## What Is Maternal Immunization?

Every pregnant woman passes on antibody to her unborn child via the placenta and through this pregnancy-associated natural phenomenon provides passive protection against a range of pathogens, simply as a ‘gift of nature.’ In a sense, each pregnant woman already naturally ‘immunizes’ her unborn child during the pregnancy, in order to protect it from pathogens during the first few months of life, as it transitions from the relatively sterile *in utero* setting to the new reality of an environment full of germs: some hostile, some not. The principle of maternal immunization essentially represents the augmentation of specific antibodies against organisms of high pathogenicity to the newborn, throughout this period of significant immune vulnerability; actively immunizing a mother during her pregnancy capitalizes on the indirect protection that will already be conferred to her newborn, thereby combining nature with nurture (Munoz and Jamieson, 2019; Jarvis et al., 2020). Maternal vaccines also have the potential to protect against congenital transmission of pathogens and viral seeding of the placenta, a strategy currently being explored primarily in the context of cytomegalovirus (CMV) (Schleiss et al., 2017; Permar et al., 2018) and herpes simplex virus (HSV) (Okala et al., 2019; Patel et al., 2019a, 2020). Risk of intrauterine viral transmission is generally higher if primary infection occurs during pregnancy, indicating that fetal protection is likely achieved through transplacental transfer of neutralizing maternal antibodies.

Table 1 briefly summarizes the vaccines recommended for (A) routine use in pregnancy, (B) use in emergencies and outbreak situations and (C) vaccines in advanced clinical development.

**TABLE 1:** Summary of vaccines recommended by WHO for **(A)** routine use by national programs, **(B)** use in emergencies or outbreak situations, and **(C)** in advanced clinical development.

Disease	Vaccine type	Recommendation
**(A) Routinely recommended vaccines for pregnant women**		
Tetanus	Toxoid	Tetanus toxoid vaccination is recommended for all pregnant women, depending on previous tetanus vaccination exposure, to prevent neonatal mortality from tetanus (World Health Organization, 2018c).
Influenza A	Inactivated	WHO recommends seasonal influenza vaccination to pregnant women as the highest priority (World Health Organization, 2020).
Pertussis	Subunit adjuvanted	Vaccination of pregnant women is likely to be the most cost-effective additional strategy for preventing pertussis disease in infants too young to be vaccinated (World Health Organization, 2016b).
**(B) Vaccines specifically recommended in endemic countries or during outbreaks**		
Cholera	Inactivated	Pregnant and lactating women should be included in Oral Cholera Vaccine campaigns. Evidence indicates high potential benefit and minimal risks (Moro and Sukumaran, 2017; World Health Organization, 2018a).
Ebola	Non-replicating or replication-deficient	Since the three new candidate vaccines are non-replicating or replication deficient, pregnant and lactating women should be included into the clinical trial protocols (UN News, 2019; Sage Interim Recommendations on Vaccination against Ebola Virus Disease, 2019).
Hepatitis E	Recombinant, adjuvanted	The use of the vaccine to reduce or prevent outbreaks of hepatitis E should be considered as well its use to mitigate consequences in high risk groups such as pregnant women (World Health Organization, 2016a).
Meningitis A (MenA)	Conjugated	Pregnant and lactating women residing in the meningitis belt receive the MenA conjugate vaccine during any stage of pregnancy or lactation (World Health Organization, 2018d).
Rabies	Inactivated	Rabies vaccines and rabies Immunoglobulin are safe and effective in pregnant and lactating women (World Health Organization, 2018b).
Tick-borne encephalitis	Inactivated	The vaccine should be used in pregnant women who live in areas where the incidence of the disease is high (>5 cases/100,000 population per year) (World Health Organization, 2011).
Yellow Fever (YF)	Live attenuated	In areas where YF is endemic, or during outbreaks, the benefits of YF vaccination are likely to far outweigh the risk of potential transmission of vaccine-related virus to the fetus or infant (World Health Organization, 2015).
**(C) Vaccines for specific use in pregnancy and in advanced clinical trials but not yet licensed or pre-qualified**		
Group B streptococcus (GBS)	Conjugated	For exclusive use in pregnancy to prevent early and late onset GBS infection in the neonate, potential impact on premature birth and stillbirths, several candidates under development. For GBS vaccine research and development technical roadmap and WHO Preferred Product Characteristics please see https://www.who.int/immunization/research/development/ppc_groupb_strepvaccines/en/
Respiratory Syncytial Virus (RSV)	Subunit +/− adjuvanted	To prevent severe RSV disease in young infants, several candidates under development. For priority activities for development, testing, licensure and global use of RSV vaccines, with a specific focus on the medical need for young children in low- and middle-income countries please see: https://www.who.int/immunization/research/development/ppc_rsv_vaccines/en/

* Most WHO vaccine position papers are easily accessible via this link: https://www.who.int/immunization/documents/positionpapers/en/.

## What Do We Know About Vaccine Responses in Pregnancy and Maternal Antibody Transfer?

### Mechanism of Transplacental Transfer

The human feto-maternal interface is complex and unique but remains incompletely understood. Immune interactions are tightly controlled in order to establish a favorable immunological environment for the developing fetus which expresses ‘foreign’ paternal antigens with the potential to activate the maternal immune system; given this, regulatory mechanisms are induced that effectively promote immunological tolerance (Jennewein et al., 2017; Than et al., 2019).

In particular, the placenta acts as an important physical and biochemical/immunological barrier between maternal and fetal circulations. It consists of the chorionic villous unit: multinucleated syncytiotrophoblast (in direct contact with maternal blood), the villous stroma and the fetal capillary endothelium. Signaling across the placenta is now considered to be bidirectional, involving both active and passive mechanisms, with a potentially significant role played by extracellular vesicles and seeding of genetically foreign maternal and fetal cells, closely regulated throughout pregnancy to ensure optimal fetal growth and development (Holder et al., 2016; Rice et al., 2018).

Maternal immunoglobulin G (IgG) is transported across the placenta from around 13-weeks’ gestation and provides passive protection to the infant in the early, most vulnerable postnatal period, prior to initiation of the primary immunization schedule (Simister, 2003; Edwards, 2015). It is an active, pH-dependent process, predominantly mediated through binding of the IgG Fc portion to the neonatal Fc receptor (FcRn) in the placental syncytiotrophoblast (van den Berg et al., 2010; Palmeira and Carneiro-Sampaio, 2016; Pyzik et al., 2019). This interaction is unique, and its significance has only recently been established (Roopenian and Akilesh, 2007; Rath et al., 2013; Jennewein et al., 2017).

Unlike syncytiotrophoblast cells, stroma or fetal endothelium appears not to express FcRn and, therefore, the mechanisms underlying the onward transport of IgG beyond the syncytiotrophoblast require further exploration; alternative non-canonical Fcγ receptors, such as FcγRI/RII/RIII, are known to be expressed by these final placental layers and may play a key role (Simister, 2003; Martinez et al., 2018).

In some cases, however, maternal IgG may facilitate vertical pathogen transmission, by forming antibody-antigen complexes which are transported across the syncytiotrophoblast via FcRn and epidermal growth factor receptor (EGFR) (Pereira and Maidji, 2008). Congenital CMV risk, for example, is greatest in the third trimester, potentially correlating with highest expression of FcRn and EGFR (and hence peak of transplacental IgG transfer). Further assessment using an *ex vivo* human placental model demonstrated that the functionality of CMV antibody may play a significant role: weakly neutralizing compared to potently neutralizing monoclonal IgG facilitated placental CMV infection (Maidji et al., 2006; Permar et al., 2018).

### Mechanism of Antibody Transfer Through Breast Milk

After birth, the placental barrier protection is replaced by the mammary gland barrier. The specific antibodies generated are also secreted into breast milk (primarily colostrum) with secretory IgA (sIgA) as the predominant antibody class, and transferred orally to infants during lactation (Schlaudecker et al., 2013; Abu Raya et al., 2014; Maertens et al., 2014; De Schutter et al., 2015). IgA is transported across alveolar epithelial cells by the polymeric Ig receptor (pIgR) and released at the apical surface (Palmeira and Carneiro-Sampaio, 2016; Jennewein et al., 2017). The extracellular domain of pIgR is the secretory component which is covalently attached to IgA, protecting it from degradation by host and microbial proteases. IgA also binds to Fc alpha receptor on the surface of myeloid cells (Aleyd et al., 2015). Recent preliminary findings, in the context of 13-valent pneumococcal conjugate vaccine given during pregnancy, also suggest that breast milk antibodies, boosted by maternal immunization, may impact the presence of vaccine antigen-specific memory B cells in Colostrum (Munoz et al., 2018).

By preventing epithelial adhesion and neutralizing toxins or virulence factors, sIgA inhibits invasion and damage from pathogens at mucosal surfaces (Maertens et al., 2014; Edwards, 2015). Interestingly, sIgA does not activate the complement cascade, contributing to anti-inflammatory benefits of breast feeding. As well as passive protection, breast milk is now considered to have an active immunomodulatory effect, promoting gut barrier homeostasis and microbiome maturation, helping to establish immune tolerance in the early postnatal period, and potentially shaping systemic infant responses; many details, however, particularly the contribution of sIgA, are still poorly understood (Oddy, 2002; Andreas et al., 2015).

### Factors Affecting Maternal Antibody Transfer Across the Placenta

In general, given the usual half-life of immunoglobulins, higher maternal and cord antigen-specific IgG concentrations are associated with subsequently longer, more effective protection in the infant in early life (Palmeira et al., 2012; Niewiesk, 2014; Fouda et al., 2018). The aim of maternal immunization is, therefore, to boost these levels well above the putative threshold of protection. Both intrinsic and extrinsic factors play a role in regulating production of adequate titres of initial maternal antibody but also its subsequent efficient transfer across the placenta. Emerging data suggests this may be selective and differentially mediated (Marchant et al., 2017; Wilcox et al., 2017). Determining the mechanisms that regulate IgG Fc-mediated functional activity at the placental interface will enable optimization of maternal vaccines in the future (Mahan et al., 2016).

#### IgG Subclass

IgG characteristics that impact FcRn interactions play a role in placental IgG transfer efficiency (Palmeira et al., 2012; Lozano et al., 2018). The IgG subclass distribution across different antigen-specific IgG populations is distinct and modulates their placental transfer efficiency, as previously demonstrated for pertussis, diphtheria, tetanus, *Haemophilus influenzae type B* (*HiB*), *Neisseria meningitidis* C and varicella zoster virus (VZV) (van den Berg et al., 2010; Vidarsson et al., 2014; Martinez et al., 2019). Specifically, IgG1 is most efficiently transferred, followed by IgG3 and IgG4, in comparison to IgG2 which is significantly reduced (Einarsdottir et al., 2014; Vidarsson et al., 2014). One contributing factor to these differences may be the IgG hinge region, which varies in its length and flexibility across subclasses, potentially impacting the orientation and movement of the Fab arms relative to the Fc tail (Vidarsson et al., 2014; Abdiche et al., 2015). It has also been reported that individual receptor types (FcRn vs. FcγRII vs. FcγRIII) may vary in their affinity and specificity for different IgG subclasses (Bruhns et al., 2009).

#### IgG Antigen-Specificity

Placental transfer is distinctly influenced by different antigen-specific IgG populations (van den Berg et al., 2010; Palmeira et al., 2012). Vaccines that contain protein antigens, such as tetanus toxoid or pertussis toxin, are transferred more efficiently than polysaccharide vaccines, including HiB and pneumococcus. The basis for this difference is unknown but may be related, in part, to subclass, given that protein antigens predominantly elicit IgG1 and IgG3 secretion, while IgG2 is more critical for the opsonisation and killing of polysaccharide-encapsulated pathogens (Vidarsson et al., 2014).

#### IgG Glycosylation

IgG exists in a number of glycosylated variants, which have undergone covalent addition of different sugar moieties and may vary in their kinetics, binding affinity to different placental Fc receptors, efficiency of transplacental transfer and functionality (Lofano et al., 2018; Jennewein et al., 2019). Fc region fucose glycans, for example, have been shown to mediate binding strength to FcγRIIIa *in vitro* (Okazaki et al., 2004; Mahan et al., 2016). Importantly, different vaccines and/or infective pathogens elicit distinct antigen-specific IgG Fc region glycan profiles (Mahan et al., 2014, 2016; Vestrheim et al., 2014). Therefore, while subclass selection alters the Fc domain irreversibly, modifying antibody glycosylation provides a more flexible mechanism by which the humoral compartment streamlines antibody effector function to effectively target a particular pathogen (Vestrheim et al., 2014; Mahan et al., 2016; Alter et al., 2018).

A recent study used an unbiased, systems serology approach (Chung and Alter, 2017) to evaluate differences in qualitative antigen-specific Fc-profiles between maternal and cord blood: skewing toward natural killer (NK) cell-activating antibodies was demonstrated in the latter across multiple antigens. This selective transfer was linked to digalactosylated Fc-glycans of antigen-specific IgG1 antibodies that show enhanced binding to FcRn and FcγR3A on NK cells. This may suggest an evolution of the placenta to selectively transfer antibodies with the most functional potential in the neonatal immune context, boosting protection, particularly anti-viral defense, in early life (Jennewein et al., 2019).

Therefore, establishing antibody glycosylation patterns associated with clinically relevant outcomes could inform the design of the next generation of improved maternal vaccines (Lofano et al., 2018; Rice et al., 2020). Equally, immunization itself is an optimal model to interrogate glycosylation patterns, gestational imprinting as well as *in vivo* regulation and persistence of glycosylation mechanisms (Alter et al., 2018).

#### Infant Gestational Age and Birthweight

The degree of IgG transplacental transfer is dependent on duration of gestation, with minimal transfer in the first trimester, increasing exponentially as pregnancy progresses, particularly in the last 4 weeks. At term, fetal levels vary but usually exceed maternal levels by 20–30%, indicating active transfer (van den Berg et al., 2011; Calvert and Jones, 2017). This change in rate of transplacental transfer may partly occur due to higher FcRn expression with advancing gestation, although this is yet to be formally demonstrated; another hypothesis is that cytotrophoblast may initially obstruct transfer, leading to improved transfer as this layer degrades (Palmeira et al., 2012; Calvert and Jones, 2017).

The reduced duration and efficiency of transfer in early pregnancy has implications for preterm infants; nevertheless this cohort has been shown to successfully benefit from maternal immunization programs (Baxter et al., 2010; Omeñaca et al., 2018; Nieminen et al., 2019; López-Sanguos et al., 2019). Interestingly, IgG Fc glycosylation patterns of infants also depend on their gestational ages; indeed, there is a qualitative shift toward a pro-inflammatory pattern in preterm infants that might contribute to their higher risk of chronic inflammatory diseases (Twisselmann et al., 2018).

Studies have demonstrated reduced transfer of antibodies in low birthweight infants, even those born at term. This may be attributed to pathological placental changes associated with intrauterine growth retardation, such as syncytiotrophoblast knotting, villous fibrosis and avascular terminal villi, affecting the antibody-FcRn interactions (Wesumperuma et al., 1999; Okoko J. B. et al., 2001). Interestingly, Wesumperuma et al. (1999) also found that iron-deficiency anemia in Sri Lankan mothers reduced placental antibody transfer. Maternal age, parity, and type of delivery was not shown to have a significant impact (Doroudchi et al., 2003).

#### Maternal Co-morbidities

Maternal co-morbidities may lead to both reduced antibody production and failure of placental integrity and key tolerance mechanisms; definitive conclusions, however, are precluded by the heterogeneity of study methodologies and findings.

It is well established that serological responses to vaccines are attenuated and wane more quickly in HIV-positive populations; the impact of viral load, immunological status and anti-retroviral therapy (ART) is important albeit inconsistent between studies (Kernéis et al., 2014; Dangor et al., 2017; Falconer et al., 2018). Comparisons between HIV-infected and -uninfected women in different settings have also shown that the former group have lower baseline/pre-vaccination protective maternal antibody levels to key vaccine pathogens and impaired transplacental transfer of IgG; this includes tetanus, GBS, VZV, measles, Hib, pertussis, and pneumococcus antibodies, although it is not a universal finding (Isabel de Moraes-Pinto et al., 1996; Cumberland et al., 2007; Jones et al., 2011, 2013; Gupta et al., 2014; Dangor et al., 2015; Le Doare et al., 2015). This may be explained by a loss of epitope-specific T- and B-memory cells secondary to immunosuppressive progression of HIV-infection (Wheatley et al., 2016; Dangor et al., 2017). Findings from influenza vaccine immunogenicity studies demonstrated that HIV-infected pregnant women had decreased vaccine-induced haemagglutination-inhibition antibody titres and a reduced likelihood of seroconversion compared to HIV-uninfected women; better responses were associated with higher CD4+ T-cells but no correlation was found with viral load (Madhi et al., 2014; Nunes et al., 2015; Dangor et al., 2017). In a separate study, altered binding to Fc receptors, FcγRIIa, and FcγRIIIa, in addition to glycan changes in the Fc region, were proposed to contribute to impaired placental integrity (Martinez et al., 2019).

Moreover, HIV-exposed uninfected (HEU) infants have up to fourfold higher rates of morbidity and mortality from diarrhoeal and respiratory infections compared to uninfected unexposed (HUU) infants, in part explained by lower maternal production and reduced placental transfer of protective IgG (Richard, 2004; Jones et al., 2011; Dauby et al., 2016; Evans et al., 2016; Slogrove et al., 2016; Locks et al., 2017; Weinberg et al., 2018). Vaccination in pregnancy could be particularly useful in improving immunity and clinical outcomes of these vulnerable infants, provided that highly functional, long-lasting antibody can be generated and transferred by their immunized mothers (Jones et al., 2013). Optimizing ART in HIV-positive women of child-bearing age may go some way toward achieving this goal, although it is unclear if reversal of adverse effects (i.e., lower baseline antibody levels and poorer booster responses to vaccines) and/or complete immune reconstitution is possible (Burton et al., 2008; Farquhar et al., 2009; Jones et al., 2013; Dangor et al., 2017). Indeed, given that HIV-infected women in LMICs are often only initiated on ART during pregnancy, loss of antigen-specific T- and B-cell memory may already have occurred. It has to be assumed that immune-status may only be preserved by early ART initiation (Moir et al., 2010).

With several candidate RSV and GBS vaccines currently in development, an improved understanding of the effects of maternal HIV infection is needed to inform vaccination strategies in areas with a high HIV prevalence and irrespective of ART use. Maternal HIV infection has been associated with lower anti-GBS surface binding antibody concentration and antibody-mediated C3b/iC3b deposition onto GBS bacteria of serotypes Ia, Ib, II, III, and V (Le Doare et al., 2015). Furthermore, immunogenicity of a CRM_197_-conjugated trivalent GBS vaccine was found to be lower in HIV-infected pregnant women compared to HIV-uninfected women, irrespective of CD4+ T-cell counts (Heyderman et al., 2016). Similarly, Patel et al. (2019b) recently showed that maternal HIV infection was associated with lower mother-to-fetus transfer of serum RSV-neutralizing antibodies. Among HEU newborns, higher birth weight and an undetectable maternal antenatal viral load were significantly associated with more effective placental transfer of RSV antibodies (Patel et al., 2019b). Nevertheless, validated assays and correlates of protection are needed to understand the potential protective value of these vaccines.

Women with other congenital or acquired immunodeficiencies, such as common variable immunodeficiency, or taking immunosuppressive medications, also have impaired serological responses, reducing production and transplacental transfer of immunoglobulins from those mothers to their offspring (Palmeira et al., 2012).

Beyond immunodeficiency, previous studies have demonstrated that placental malaria impairs transplacental transfer of different antigen-specific IgG populations due to parasitic damage to the villi architecture described earlier. This has been shown in the context of tetanus, measles, *Streptococcus pneumoniae*, HSV-1, EBV, RSV, and VZV antibodies (Brair et al., 1994; Isabel de Moraes-Pinto et al., 1996; O’Dempsey et al., 1996; Okoko B. J. et al., 2001; Cumberland et al., 2007; Ogolla et al., 2015) although results are conflicting, potentially due to differences in the study population and laboratory assays used (Calvert and Jones, 2017). Subsequent data from malaria-endemic Papua New Guinea proposed that the association previously reported between malaria and impaired RSV antibody transfer and/or reduced RSV cord titres, may have been confounded by prematurity or hypergammaglobulinemia (IgG > 1,700 mg/dL) (Atwell et al., 2016, 2019). In fact, the impact of high total IgG concentrations on placental IgG transfer efficiency has previously been shown (Gonçalves et al., 1999; Hartter et al., 2000; Okoko B. J. et al., 2001) in both HIV-infected and -uninfected populations (Martinez et al., 2019). The underlying mechanism is unknown but likely due to saturation of nFcRs (Englund, 2007; Fouda et al., 2018).

Chronic helminth infections during the time of vaccination are thought to impair the induction and duration of protective immune responses elicited by vaccines (Dauby et al., 2012; Gent et al., 2019) potentially contributing to attenuated vaccine responses observed in pregnant cohorts in developing countries where helminth infections are endemic (Sabin et al., 1996; Elias et al., 2005; Riner et al., 2016).

Other infectious or inflammatory conditions, including hypertension, hyperglycaemia and placental pathologies (e.g., preeclampsia), may alter IgG production, damage placental villi, decrease FcRn expression and/or compromise transplacental transfer rate (França et al., 2012; Predoi et al., 2015; de Souza et al., 2016; Fouda et al., 2018). Of note, however, these conditions are also associated with prematurity and intrauterine growth retardation (Palmeira et al., 2012). There are no specific studies relating to maternal vaccination in these risk groups.

### Factors Affecting Maternal Antibody Transfer in Breast Milk

Similar factors may contribute to maternal antibody transfer in breast milk, although there is paucity of data. One example is the impact of vaccination timing and gestational age; significantly more sIgA (and IgG) was measured in the colostrum and mature breast milk of women who delivered preterm, waning more gradually than in term women (Araújo et al., 2005; Ballabio et al., 2007). This may be an immunological adaptive response boosting protective immunity to vulnerable preterm infants (Gregory and Walker, 2013). Survival and stability of milk antibodies is also higher with prematurity (Demers-Mathieu et al., 2018).

Equally, IgA Fc region characteristics may determine IgA passive transfer or effector function in breast milk (Goonatilleke et al., 2019; Langel et al., 2020; Steffen et al., 2020). Of note, the glycosylation pattern of IgA antibodies is more complex, extensive and diverse; IgA2 is found at a higher percentage in mucosal secretions and has a great number of conserved *N*-glycans compared to IgA1, which dominates in serum (Mattu et al., 1998). *N*-glycan profiles may also be influenced by delivery mode (Goonatilleke et al., 2019). Further defining the molecular determinants of antibodies in breast milk, and whether they complement placentally derived antibodies, may enable streamlining of breast milk immunity through maternal vaccination and other postnatal strategies (Kollmann et al., 2020).

### Beyond Maternal Antibody

To date, the goal of immunization in pregnancy has primarily been to induce robust maternal antibody responses. Nevertheless, robust vaccine-elicited maternal T-cell responses may also be required to ensure complete protection, particularly against transplacental pathogen transmission and subsequent congenital disease. In the context of CMV, for example, T-cell responses may contribute to eliminating virus-infected cells and supporting B-cell responses, with CMV-specific CD4+ T-cell frequency and/or proliferation playing a critically important role in preventing transmission during pregnancy (Revello et al., 2006; Lilleri et al., 2007; Fornara et al., 2016). This may need to be harnessed if an effective maternal vaccine is to be developed.

In addition, maternal cells are increasingly thought to migrate transplacentally to offspring at low frequencies (Kinder et al., 2015). Further pathogen-specific immunity may, therefore, be conferred to the newborn, enhancing protection already delivered by maternal antibody (Albrecht and Arck, 2020; Kollmann et al., 2020). This is based on the principle that bidirectional transfer can occur during pregnancy, with seeding of genetically foreign maternal and fetal cells, known as ‘microchimeric cells.’ The biological function and molecular phenotypes of these rare fetal (FMC) and maternal (MMC) microchimeric cells is poorly understood. It is hypothesized that they may help establish immunological tolerance to an expanded repertoire of familially-relevant ‘extended-self’ antigens, as well as promote the success of future pregnancies by conferring cross-generational reproductive benefits. The underlying principles, immunological implications, potential advantages and harmful consequences of microchimerism have been reviewed extensively elsewhere (Kinder et al., 2015, 2017a; Jennewein et al., 2017).

The MMCs express non-inherited maternal antigens and can persist in the offspring long-term, detectable even up to 62 years postnatally. A considerable proportion belong to the immune compartment, particularly T-cells or tissue-resident memory cells (Albrecht and Arck, 2020); it is estimated that up to 1 in 5,000 peripheral blood mononuclear cells may be of maternal origin (Kinder et al., 2017a). In a previous case of a human infant with severe combined immunodeficiency, activated CD8^+^ T-cells and IFN-γ-secretion were detected in response to EBV infection; these cells displayed a maternal genotype (Touzot et al., 2012). Furthermore, using a mouse model, a recent study established that non-inherited maternal antigen-specific regulatory T-cells are acquired in early life and persist in the genital tract of female offspring, thereby supporting a role for MMCs in the immunological development of infants and cross-generational reproductive fitness (Kinder et al., 2015).

Microchimerism is less well-established in the context of breast milk; animal experiments and limited human-based observations suggest that maternal immune cells can be detected in breast milk and may traffic to infant tissues through gut mucosae (Palmeira and Carneiro-Sampaio, 2016; Kinder et al., 2017b; Molès et al., 2017). This phenomenon most likely occurs during the early stages of lactation, primarily colostrum, when breast milk cell abundance and infant gut permeability are highest. These MMCs may include stem cells, progenitor cells and/or mature immune cells (such as IgG-producing memory B-cells and memory T-cells), although this remains poorly understood (Kinder et al., 2017b; Marchant et al., 2017). Interestingly, a recent murine study demonstrated that, under the same activation conditions, maternally-derived CD8+ T-cells in breast milk are superior in generating potent mediators compared to the infant’s endogenous cellular compartment. This may be a compensatory mechanism for the infant’s unique adaptive immune system during the vulnerable postnatal phase (Cabinian et al., 2016). Molès et al. (2017) hypothesize, therefore, that the transfer of viable maternal immune and stem cells from breast milk to an infant may contribute to optimizing neonatal and infant immune system maturation, cross-generational reproductive fitness, tissue repair and immune tolerance, thereby complementing pregnancy-associated MMCs (Molès et al., 2017). However, this hypothesis has been challenged and the exact mechanisms are yet to be fully elucidated, particularly in the context of vaccine-induced maternal leukocyte transfer (Kinder et al., 2017b).

## So, Is There a Right Time to Vaccinate?

The optimal timing of vaccination in pregnancy remains debated and the kinetics will have to be considered when strategically deploying maternal vaccination to maximize impact in the future (Calvert and Jones, 2017). A recent review of prevalence and decay of maternal antibodies from different pneumococcal and meningococcal vaccine trials demonstrated differences between serotypes, serogroups, and countries (Voysey et al., 2017). Moreover, many countries advise to immunize against pertussis at every pregnancy, since antibodies rapidly wane after an adult booster dose and decline in the infant after birth, mostly within 2 months (Halperin et al., 2011; Healy et al., 2013).

Vaccination in the first trimester is usually avoided as this trimester is associated with higher risk of pregnancy loss and the time of major fetal organogenesis. Approaches that favor third trimester immunization point to the relatively short half-life of vaccine-induced antibody, efficiency of placental transfer as gestation advances and the need to match the highest antibody levels with the peak of transplacental IgG transport (Healy et al., 2013; Naidu et al., 2016; Winter et al., 2017). Vaccinating earlier in pregnancy, however, is likely to provide better protection for preterm infants. Eberhardt et al. (2016) proposed that maternal pertussis immunization in the second trimester maximized antibody transfer and expected infant seropositivity against pertussis, potentially because antibody can accumulate over a longer time period following earlier vaccination. Furthermore, timing may impact antibody avidity in cord blood, with a recent study demonstrating that newborns of women receiving pertussis vaccinations between 27 and 30 + 6 weeks gestation had a higher relative avidity index than those of mothers vaccinated later (Abu Raya et al., 2015). Nevertheless, a remainder of studies have shown no significant relationship between timing of maternal vaccination and subsequent antibody levels in cord/infant blood or proportions achieving protective thresholds for most antigens (Ladhani et al., 2015; Vilajeliu et al., 2015), hence these kinetics might differ between vaccines and the respective antibody levels they can induce.

## Once Transferred, What Do We Know About the Effect of Maternal Antibody on the Newborn Immune System?

### Systemic Infant Humoral Immunity

One of the main controversies in the field of maternal immunization is whether high maternal vaccine-induced antibody titres interfere with or ‘blunt’ the infant’s endogenous antibody response following primary immunization and, therefore, potentially threaten effective disease protection. Blunting has been demonstrated previously in the context of pertussis, influenza, tetanus, diphtheria, measles, and mumps immunity, hence it is an important consideration for future maternal vaccine development and implementation (Englund, 2007; Jones et al., 2010, 2014; Ladhani et al., 2015; Maertens et al., 2017; Zimmerman et al., 2019; Orije et al., 2020).

Several mechanisms have been proposed to explain an inhibitory effect of maternal antibody on infant B-cell responses: (1) antigen neutralization of live replicating viral vaccines (2) epitope masking preventing antigen binding by infant B-cells, thereby limiting their priming, the most popular theory (3) inhibition of infant B-cell activation by FcγRIIB-receptor-mediated signaling, and (4) removal of maternal antibody-vaccine antigen immune complexes by Fc-dependent phagocytosis (Siegrist, 2003; Kim et al., 2011; Niewiesk, 2014; Edwards, 2015).

Although a blunting response has been demonstrated, this data is generated from immunogenicity studies and, to date, there is no evidence of relevance to clinical outcomes (disease incidence or severity) (Kandeil et al., 2020); however, this is difficult to ascertain, particularly with vaccines that have no definitive correlate of protection (CoP). Despite the association between low anti-PT IgG titres and high susceptibility to pertussis disease, a protective antibody threshold is yet to be established (Storsaeter et al., 2003). Nevertheless, surveillance data collected in settings where maternal pertussis immunization programs have been implemented show a highly successful reduction in disease morbidity and mortality in early life (Amirthalingam et al., 2014, 2016; Switzer et al., 2019; Forsyth et al., 2020; Jarvis et al., 2020).

Furthermore, the blunting effect noted has not been comprehensively defined, with a gap in our understanding of the impact of maternal antibody on qualitative or functional infant humoral immunity. Pre-existing antibodies have been shown to induce higher affinity humoral responses, possibly explained by competitive binding in the germinal center (GC) and/or a boost in uptake and antigen presentation, mediated by immune complexes in a Fc glycosylation–dependent manner (Zhang et al., 2013; Lofano et al., 2018; Garg et al., 2019; Langel et al., 2020). Most recently, a randomized control trial in Thailand (NCT02408926) studied term infants who were immunized with either acellular (aP)- or whole cell (wP) pertussis vaccines in infancy; results showed that infants born to women vaccinated in pregnancy had reduced pertussis-specific titres. Despite this, antibody functionality (as determined by *B. pertussis* growth inhibition assay) was overall better in wP-vaccinated infant sera, and after maternal immunization. These data suggest that maternal antibodies may boost the production of antibodies with distinct biophysical features to achieve effective pathogen control in the infant (Wanlapakorn et al., 2019).

Finally, many have argued that blunting is short-lived and reversible, especially as maternal antibodies decline over time, and does not remain significant following booster vaccination (Munoz et al., 2014; Le Doare et al., 2015; Hoang et al., 2016). Longitudinal follow-up studies, therefore, are required to arrive at stronger conclusions on the clinical implications of blunting, if any, potentially also in the context of different infant immunization schedules and environmental exposures. Such studies are hard to do in the absence of meticulous long-term follow-up of infants born to vaccinated mothers, at the population level.

### Systemic Infant Cellular Immunity

To date, vaccine-induced CoPs are predominantly defined by the development of serum antibody responses using quantitative assays, with less focus on cell-mediated immunity (CMI), despite the significant induction of cellular responses post-vaccination by some vaccines, such as pertussis, and the important role CMI plays in disease protection (Ausiello et al., 1999; da Silva Antunes et al., 2018; Poland et al., 2018; Wilk and Mills, 2018). This extensive knowledge gap is likely due to the practical and biological difficulties of accurately and reliably measuring CMI. Assays are costly, challenging, labor-intensive, often requiring large amounts of blood and, above all, poorly standardized, limiting comparisons between different studies (Wilcox and Jones, 2018).

A lowered antibody titre, caused by the blunting effect, does not necessarily imply reduced protection; a recent comprehensive review concluded that in the majority of both human and animal studies, priming of CMI after infant vaccination occurred even in the presence of high maternal antibody titres, with minimal or no blunting effect on cellular responses reported (Orije et al., 2020). Furthermore, in two studies, maternal antibodies were even found to stimulate a more robust CMI response, highlighting a potential secondary beneficial effect of maternal immunization (Bertley et al., 2004; Rowe et al., 2005).

In contrast to other blunting theories, a recent study by Vono et al. (2019) showed that maternal antibodies do not prevent neonatal B-cell activation but exert their influence by limiting the expansion of T-follicular helper cells, thereby shaping GC output, i.e., B-cell differentiation into effective plasma cells and/or memory cells, and the antigen-specific B-cell repertoire (Vono et al., 2019). Interestingly, at low or intermediate titres, maternal antibodies did not prevent the induction of memory-cells, suggesting a gradient effect on infant immune responses (Vono et al., 2019; Langel et al., 2020).

#### *In utero* Sensitisation and Priming

There is now a growing body of evidence that the fetal immune system may be shaped by vaccination through more than just the passive immunity provided via IgG transfer. One potential mechanism, discussed previously, is maternal microchimerism, which is due to transplacental bi-directional migration of cells and may play a role in neonatal immune modulation (Kinder et al., 2017a). Furthermore, as demonstrated already in the context of infectious disease antigens (Bisseye et al., 2009; Malhotra et al., 2009; Dauby et al., 2012; Zhivaki and Lo-Man, 2017; Odorizzi et al., 2018) the fetus may also be sensitized *in utero*, both qualitatively and quantitively, to vaccine antigens to which the mother has encountered during pregnancy, either as free antigens or antigen-antibody complexes (or, potentially, antigen-loaded vesicles, Tong and Chamley, 2015). This exposure during fetal development has previously been shown to imprint immunological tolerance to non-inherited ‘foreign’ maternal antigens, persisting beyond early infancy into adulthood (Kinder et al., 2015, 2017a; Jennewein et al., 2017).

Previous studies of maternal immunization have identified antigen-specific IgM in cord blood, assumed to be secondary to activation of fetal B-cells, given that minimal IgM crosses the placenta; this has been demonstrated following influenza and tetanus vaccination, although there is little data following pertussis immunization (Gill et al., 1983). Rastogi et al. (2007) detected antigen-specific T-cells (more directly, using MHC Class I tetramers) in the cord blood of infants born to influenza-vaccinated compared to non-vaccinated mothers; their phenotype was CD45RO+, indicating an effector memory T-cell response, usually not demonstrated in cord blood, and therefore proposed to be secondary to vaccine-induced *in utero* priming (Rastogi et al., 2007). Such memory T-cells display various effector functions, including Th1, Th2, or Th17 profiles (Zhivaki and Lo-Man, 2017). Other studies, however, have challenged that these proposed antigen-specific fetal T-cells are conventionally primed memory T-helper cells; instead, they may represent ‘recent thymic migrants,’ a common cell population in the neonatal cohort and a transitional subtype between thymocytes and adult T-cells (Wilcox and Jones, 2018). More research in this area is required and a systems approach might be needed to better understand the complexities and interdependencies.

The potential clinical relevance of these findings also remains unclear. Vaccine-induced *in utero* generation of T-cell memory could benefit the neonate by shaping immune ontogeny and conferring pathogen-specific protection, beyond antibody-mediated passive immunity (or Th2-skewed responses). Moreover, it may be relevant in cases where the pediatric primary immunization course is too late to prevent severe disease, for example, with GBS or RSV infections in early life (Zhivaki and Lo-Man, 2017). The possible impact on responses to childhood vaccines must also be explored.

#### Heterologous (Non-specific) Effects

Another recent concept with relevance to the impact of vaccination in pregnancy on the mother, fetus or newborn is the notion of heterologous or ‘non-specific effects’ (NSE) of vaccines, including heterologous lymphocyte effects and induction of innate immune memory (‘trained immunity’). The latter is thought to be mediated by epigenetic and metabolic reprogramming which elicits long-term functional upregulation of innate immune cells (Netea and van der Meer, 2017; de Bree et al., 2018). NSE are hypothesized to explain findings by some studies that certain vaccines have a broader impact on health outcomes than previously appreciated; protection may be conferred against unrelated pathogens, beyond those specifically targeted by the original vaccine design (Aaby et al., 2010; Saadatian-Elahi et al., 2016; Pollard et al., 2017; de Bree et al., 2018; Uthayakumar et al., 2018). This is currently an under-explored area of research within the mother/infant dyad, although one recent study did show that maternal MF59-adjuvanted influenza immunization was associated with an altered cytokine profile in the nasal mucosa of 4-week-old infants subsequently, when compared to those born to unvaccinated mothers (Bischoff et al., 2015). This may be important when modeling the impact of programs that harness a dual maternal and infant vaccination strategy (Munoz et al., 2018).

### Infant Mucosal Immunity

In addition to preventing disease, maternal immunization may be a strategy to protect young infants from early bacterial carriage at the mucosa, via either breast milk- or transplacentally derived antibody (Chaithongwongwatthana et al., 2015). This is being explored particularly in the context of maternal pneumococcal, GBS and pertussis vaccines, whereby colonization is a prerequisite to invasive disease, although results are inconclusive and potentially confounded by maternal carriage status at birth (Munoz et al., 2001; Le Doare et al., 2017; Ojal et al., 2017). A vaccine-induced CoP may, therefore, need to be defined as a composite measure in some cases, addressing impact on carriage, infection and disease.

Similarly to transplacental antibody, we must consider whether interference from vaccine-derived antibodies in breast milk may impede immunogenicity of mucosal vaccines; it has been speculated that this may contribute to the poor performance of live oral rotavirus immunizations given to infants in LMICs, where breastfeeding rates are high (Jiang et al., 2014; Parker et al., 2018). A recent systematic review evaluating the role of maternal immunity in rotavirus vaccine immunogenicity concluded that higher levels of transplacental rotavirus-specific IgG antibody and, to a smaller extent, breast milk rotavirus-specific IgA contribute to reduced or failed rotavirus vaccine seroconversion in infants; however, clinical trials withholding breastfeeding at the time of vaccination had no significant effect on vaccine responses (Rongsen-Chandola et al., 2014; Mwila et al., 2017). Nevertheless, antibodies or other immune factors may persist in the infant’s gastrointestinal tract for longer periods than the time spent on withholding feeds. Maternal IgG/IgA antibodies have also been shown to dampen mucosal cellular responses against commensal bacteria in mice (Koch et al., 2016). Lack of a definitive CoP, however, similarly applies to breast milk studies. Even with vaccines that have a known CoP, it is unclear whether this can be extrapolated to sIgA titres in breast milk (Plotkin, 2010; Maertens et al., 2014).

## The Bigger Picture: What Else Influences the Mother/Infant Dyad of Interactions?

We have already discussed factors that may affect transfer of maternal antibody to infants, but adequate production of antibody in the first place is a crucial determinant of subsequent infant antibody titres. Further maternal, neonatal and environmental factors also shape the early immunological milieu and, therefore, the success of maternal immunization (Kollmann et al., 2017). A detailed discussion of all key factors impacting vaccine responses in general is beyond the scope of this article and has been extensively reviewed elsewhere (Zimmermann and Curtis, 2019).

### Host Factors

#### Intrinsic

It is well established that age and sex affect vaccine-induced immunity, particularly quantitative antibody responses, relevant to both the pregnant woman and her developing infant (Baxter et al., 2010; Omeñaca et al., 2018; Chiappini et al., 2019; López-Sanguos et al., 2019).

Specifically, females display enhanced immune reactogenicity, with higher antibody responses and adverse events, than males (Fischinger et al., 2019). Furthermore, immunogenetic studies have suggested that host genetic polymorphisms modulate heterogeneity in vaccine responses in the context of numerous immunizations, for example, against measles, hepatitis B, influenza A, BCG, HiB, and certain *Neisseria meningitidis* serotypes. This includes variants in the genes regulating both innate and adaptive compartments, encoding Toll-like receptors, HLA molecules, cytokines, and cytokine receptors. However, very few data are available from studies conducted in infants (Newport, 2015; Linnik and Egli, 2016).

#### Nutritional Status

The effect of nutritional status and nutritional supplements is contentious. Many studies in adults show that an elevated body mass index (BMI) is inversely correlated with long-term vaccine-induced antibody responses (Sheridan et al., 2012). By contrast, adequate maternal nourishment, particularly micronutrient intake, is important to achieve optimal vaccine responses (Wu et al., 2019); malnutrition may be exacerbated in pregnancy when nutritional demand is highest. Exposure to a nutritionally deficient environment during fetal life and early infancy may further adversely alter the ontogeny of the neonatal immune system, impacting early growth and development, including the infant’s ability to mount optimal immune responses to vaccination. A recent randomized trial in rural Gambia, the Early Nutrition and Immune Development (ENID) study, demonstrated that maternal supplementation with multiple micronutrients combined with protein-energy during pregnancy enhanced antibody responses to routine DTP vaccination in early infancy, although there were key methodological limitations (Okala et al., 2019). The mechanisms underlying these associations warrant further investigation. EMPHASIS, a study based in India and sub-Saharan Africa, aimed to characterize epigenetic features linking pre-conceptional nutrition and subsequent health-related outcomes in children, although specific vaccine responses are yet to be elucidated (Chandak et al., 2017). Investigations into the immunological benefits of vitamins, such as vitamins A, B, or D, report conflicting, inconclusive results (Sadarangani et al., 2015; Zimmermann and Curtis, 2019).

### Environmental Factors

#### Geographical Setting

Geographical setting, both defined by development status or urban/rural location, is known to play a role in immune responses at all ages and might also affect responses to vaccines in pregnancy (Zimmermann and Curtis, 2019). Equally, season and climate are important factors when considering disease burden and, therefore, practicalities of implementing vaccination programs during pregnancy, particularly with respiratory pathogens RSV and influenza (Moore et al., 2006).

#### Microbiome

Data is increasingly showing a mutualistic relationship between the intestinal microbiota and vaccine responses (Ferreira et al., 2010; Rogier et al., 2015; Gensollen et al., 2016; Nguyen et al., 2016; Lynn and Pulendran, 2018; Zimmermann and Curtis, 2018). Higher relative abundance of *Actinobacteria* and *Firmicutes* was consistently correlated with both higher antibody and cellular responses to several vaccines, including BCG, Hepatitis B, IPV, OPV, and tetanus immunizations; an inverse correlation was found for *Proteobacteria* and *Bacteroidetes* (Zimmermann and Curtis, 2018; Huda et al., 2019). There has been less focus, however, on the impact of the respiratory microbiome on vaccine responses (Mika et al., 2015, 2017; Tarabichi et al., 2015; Zimmermann and Curtis, 2018; Lee K. H. et al., 2019). One important recent study showed a positive association between nasopharyngeal colonization with *Bacteroides ovatus*, *Lactobacillus helveticus*, *Prevotella melaninogenica*, *Streptococcus infantis*, and *Veillonella dispar* and influenza virus-specific H1 and H3 IgA levels in nasal washings after vaccination with live-attenuated influenza vaccine (Salk et al., 2016). These findings are relevant when optimizing vaccine responses during pregnancy as well as assessing natural and vaccine-induced immune development of the infant. The underlying immunological mechanisms, however, have yet to be fully elucidated; changes in the level of microbially-derived metabolites may activate the innate immune compartment, thereby modulating development of T-and B-cells (Janoff et al., 2012; Lynn and Pulendran, 2018). Breast milk components, including the diverse array of microbiota, may further shape the neonatal immunological milieu, potentially mediated through human milk oligosaccharides/glycoconjugates, milk-derived sIgA/IgG and cytokines (Geuking et al., 2012; Rogier et al., 2015; Toscano et al., 2017; Kirmiz et al., 2018; Le Doare et al., 2018).

Although postnatal colonization plays a key role in setting the immune phenotype of the offspring, the process begins in early gestation. Using a model of reversible colonization of germ-free mice during pregnancy, signals derived from maternal microbiota were shown to influence early infant immune development and function, for example by maturing fetal/neonatal intestinal innate immune cells and altering intestinal gene expression profiles (De Agüero et al., 2016). In addition, metabolites that originate directly from the maternal diet, often modulated by the microbiota, can be transferred to the offspring and may potentially further shape its immunity (Macpherson et al., 2017). Maternal antibodies are critical in amplifying this transfer, both across the placenta *in utero* and during lactation, by binding and retaining bacterial products and efficiently delivering them to offspring (Ganal-Vonarburg et al., 2017). Furthermore, Koch et al. (2016) showed that mice lacking maternal antibodies display dysregulated mucosal responses.

At present, this complex interrelationship between maternal antibody, microbiome signaling, dietary metabolites and neonatal immunity is poorly understood in humans; notably, there are no current studies relating it to different maternal vaccines and/or subsequent infant vaccine responses.

### Vaccine Factors

Vaccine antigen content, dosage, adjuvant composition and route of administration will affect the quality and magnitude of immune responses both in the mother and, subsequently, her infant (Zimmermann and Curtis, 2019). In particular, modifying these factors may alter key antibody properties, related to both structure and function (Alter et al., 2018). Novel adjuvants are often added to subunit vaccines to enhance their immunogenicity, both qualitatively and quantitatively; in the context of maternal immunization, they may optimize magnitude and transplacental transfer of antigen-specific antibodies to the fetus (Francica et al., 2017). However, their potential to induce pro-inflammatory reactions and, hence, any risk of adverse reproductive, teratogenic or fetal developmental effects must first be rigorously interrogated (Herberts et al., 2010; WHO: Global Advisory Committee on Vaccine Safety, 2014).

The safety of alum-adjuvanted vaccines in pregnancy has been well established (e.g., tetanus and pertussis) and increasing evidence is emerging on oil-in-water adjuvants, such as MF59, which has shown to improve both epitope breadth and binding affinity of antibody responses if used in hemagglutinin -based influenza vaccines (Tsai et al., 2010; Rubinstein et al., 2013). Another promising example is a next-generation polysaccharide-based adjuvant developed from microcrystalline particles of delta inulin (Advax^TM^), so far shown to be safe and effective in seasonal/pandemic influenza vaccines in non-pregnant human adults, as well as in pregnant animal models (Honda-Okubo et al., 2014).

Induction of durable vaccine-specific antibodies with a distinct glycosylation pattern or from a particular subclass may be an effective strategy to optimize immunogenicity (Saunders, 2019). Modifying vaccine-type, dosing (single vs. multiple), delivery route and adjuvant formulation (to stimulate different Toll-like receptors) can selectively modify the Fc domain and induce glycan combinations which confer desired antibody effector functions (Selman et al., 2012; Mahan et al., 2014, 2016; Francica et al., 2017; Lofano et al., 2018). For example, removing the glycans from IgG2b has previously eliminated its immunosuppressive activity both *in vitro* and *in vivo* (Langel et al., 2020).

The antigen composition of the vaccine is of course also key, particularly the choice of immunogenic epitopes. Live-attenuated vaccines are generally excluded from use in pregnancy due to concerns over potential reversion to virulence and subsequent fetal complications, or the risk of adverse events in immunocompromised pregnant women. Substantial literature, however, has reported no concerning effects in the fetus following monovalent rubella, combined measles-mumps-rubella, yellow fever and oral poliovirus vaccines (WHO: Global Advisory Committee on Vaccine Safety, 2014; Laris-González et al., 2020). Nevertheless, wider conclusive evidence on safety issues is needed to inform future recommendations, as this may be an important strategy particularly with less immunogenic vaccines.

Moreover, many novel types of influenza vaccines are under development, including those that incorporate alternative proteins derived from the nucleus or M2 channel, as well as potential ‘universal’ DNA and RNA vaccines (Krammer et al., 2018; Lee et al., 2018). In the context of RSV, a recent phase 2 study of a novel RSV fusion (F) protein nanoparticle vaccine given to a large cohort of third-trimester pregnant women showed promising results, even if the primary endpoint was not met (Muňoz et al., 2019).

Increased antigen load or recurrent dosing may also be important in certain cohorts, such as immunodeficient women who cannot mount sufficiently high antibody responses to reach the protective threshold with routine doses.

Finally, future vaccine development should consider the impact of different delivery systems or immunization routes, including mucosal strategies, particularly if protection against colonization is established as an important endpoint.

Above all, pregnant women have been traditionally excluded from many vaccine trials, which has precluded sufficient good quality data on long-term maternal/fetal safety and vaccine-induced efficacy within this cohort. Fortunately, recent initiatives and multi-stakeholder involvement are beginning to turn the tide.

## Approaches to Close Our Gaps in Knowledge

### Methodology

The advent of systems vaccinology has enabled us to generate multifaceted datasets using high- throughput technologies and integrate them with sophisticated computational analysis, providing detailed insights into the effects of maternal immunization, associations between materno-fetal immunological parameters, interactions at the placental barrier and a full characterization of breast milk analytes (Nakaya and Pulendran, 2015; Poland et al., 2018; Lee A. H. et al., 2019). To date, however, our lack of standardized tools to quantify antigen-specific T-cells has hampered our ability to confirm the evidence of *in utero* sensitisation and priming of the fetal immune system following exposure to maternal vaccine antigens, and to fully characterize the impact of maternal antibody on subsequent vaccine-specific cellular responses in infants.

Novel approaches include the study of high-dimensional cell-subset immunophenotyping through CyTOF (Porpiglia et al., 2017; Lingblom et al., 2018; Reeves et al., 2018) and vaccine-induced changes at the metabolic (Li et al., 2017) proteomic (Galassie and Link, 2015) genetic and transcriptional (Stubbington et al., 2017) levels. This knowledge is largely being harnessed to establish signatures predictive of vaccine immunogenicity (Pezeshki et al., 2019), although early inflammatory transcriptomic profiling has recently been explored in the context of vaccine safety (Tregoning et al., 2020). Systems serology has also been used to define Fc features associated with antibody transfer and effector function, as discussed previously (Chung and Alter, 2017; Jennewein et al., 2019) including a detailed characterization of adjuvant effects on antibody quality (Francica et al., 2017). This could be extended to breast milk antibodies and interactions, given the lack of a validated standardized assay for sIgA.

**Box 1. Gaps in knowledge and future avenues of research in pregnant women.**

• Changes in placental structure, development and function throughout gestation, including materno-fetal regulatory mechanisms.
• Factors that determine placental integrity, successful maternal IgG-FcRn interactions, and subsequent efficacy of (bi-directional) transplacental transport.
• Impact of maternal antibody avidity on placental transfer and the impact of timing on avidity.
• Quantitative and qualitative changes in Fc glycosylation throughout pregnancy and across different antigen-specific populations, as well as the effect of pregnancy, disease and specific vaccine factors on glycosylation patterns.
• Biological and clinical implications of distinct glycosylation profiles, including potential association with Fc-mediated maternal antibody interference.
• Detailed mechanisms underlying breast milk antibody transfer and key regulating factors.
• Effects of vaccination during pregnancy on the composition of breast milk, particularly the presence of pathogen-specific sIgA antibodies.
• How vaccine design, delivery, dosing and timing may determine efficacy of antibody transfer, in the context of different types of vaccines.
• Robust experimental models to interrogate the materno-fetal interface, specifically utilizing new technologies and imaging techniques.
• Definitive (potentially composite) correlates of vaccine-induced protection for key maternal vaccines.

**Box 2. Gaps in knowledge and future avenues of research in infants.**

• Application of novel systems biology tools to investigate placental, maternal, fetal, and neonatal immune compartments.
• Standardized qualitative and quantitative assays to fully characterize the short-, intermediate- and long-term effects of maternal immunization on fetal and neonatal immunity.
• Targeted studies on the maternal, neonatal (both genetic and acquired) and environmental co-factors shaping early neonatal quantitative and qualitative immune responses.
• Mechanisms explaining blunting and its potential impact on functional responses.
• Mechanisms underlying potential maternal vaccine-induced microchimerism and *in utero* priming, plus the impact on subsequent infant cell-mediated responses to primary vaccines, including heterologous effects.
• Interactions between IgG with other Fc receptors, particularly on fetal or neonatal innate cells.
• The role of vaccine-induced mucosal cellular immunity and its interaction with mucosal humoral responses and/or microbiota communities.
• Impact of breast milk immune factors induced by maternal immunization on infant immune responses.
• Biological and clinical relevance of immune phenomena induced by maternal vaccination in the context of infant infection and immunity.

Conversely, a systems vaccinology approach can help to understand the effect of pregnancy itself on vaccination, including the specific acute inflammatory pathways induced and markers of immunogenicity/safety. Using Boostrix vaccine (combined diphtheria, tetanus, multivalent acellular pertussis), Tregoning et al. (2020) demonstrated that pregnancy might have minimal impact on initial vaccine-induced responses; they observed characteristic patterns of gene expression, including upregulation in interferon response and innate immunity gene modules that were independent of pregnancy, irrespective of baseline differences and similar in both women and mice. Given this, they propose that studies in non-pregnant women can provide information about maternal vaccine immunogenicity and potentially safety; using murine models may also be promising in this context (Tregoning et al., 2020).

Therefore, it is hoped that newer ‘omics’ assays may elucidate key mechanisms controlling vaccine immunity, particularly functional responses, define molecular signatures, identify novel CoP and predict clinical end-points, including safety. It is time to direct these tools to the maternal/infant dyad during clinical trials.

### Experimental Models

These methods must be applied within a convincing model of the materno-fetal interface. Paired maternal–cord samples, specifically the ratio of cord:maternal antibody concentration, are often used as a surrogate for placental transfer, although this fails to provide mechanistic insights.

#### Animal Models

Animal models have provided ‘biologically complete’ insights into the possible mechanisms of FcRn-mediated IgG transfer, overcoming ethical and practical limitations of human studies. Nevertheless, the morphological, developmental, physiological and immunological characteristics of the placenta often vary between humans and animals, including the expression of FcRn and mechanisms of IgG transport (Carter, 2007; Grigsby, 2016).

Similarly, animal models of breast milk production/transfer are limited; differing from humans, IgG is the primary immunoglobulin class in milk throughout many animal species (rodents, bovines, cats, and ferrets), predominantly transported across the duodeno-jejunal epithelium into the neonatal circulation (similar FcR-mediated mechanism to human transplacental transfer) (Van De Perre, 2003). The protective effect of breast milk may be proven by performing foster feeding studies, whereby offspring of immunized mothers are nursed by non-immunized mothers and vice-versa, clearly not feasible in human cohorts (Maertens et al., 2014; Grigsby, 2016).

#### Human *in vitro* Models

Freshly isolated primary term cytotrophoblasts can be cultured, differentiating into multinucleated syncytiotrophoblast cells, although there may be ethical, biological and practical constraints (Depoix et al., 2013). Alternatively, immortalized or choriocarcinoma-derived cell lines are used in directional transport and metabolic studies (Orendi et al., 2011); when combined with fluorescence microscopy of FcRn-green fluorescent protein-transfected live human endothelial cells, analysis of the intracellular trafficking of IgG is facilitated in real-time (Ober et al., 2004; Turco and Moffett, 2019). Another strategy is the placental explant model, prepared from the villous placenta (usually from early terminations) and adhering to a plastic or defined matrix. This can be used both *ex-vivo* or *in-vitro* and performed at any stage of gestation, unlike trophoblast cells or placental perfusion models, enabling interrogation of early placental function (Miller et al., 2005; Viall et al., 2013). These models have recently helped to demonstrate bi-directional extracellular vesicle-mediated transfer of proteins, lipids and nucleic acids (Tong and Chamley, 2015); exosomes from activated immune cells were shown to signal to the placental unit, contributing to materno-fetal communication, in addition to modulating placental- and possibly fetal- immunity (Holder et al., 2016; Aengenheister et al., 2018). This warrants further investigation in the context of maternal disease and vaccine antigens/immunity. Moreover, to date, application of these models to antibody investigations has been limited.

#### Human *ex vivo* Models

The gold standard for assessing placental transfer is the placental perfusion model, although it is complex and technically challenging, requiring very rapid access to fresh placenta samples (Conings et al., 2017). A placental cotyledon is cannulated and dually perfused to replicate the independent fetal and maternal circuits. This technique is simplified, primarily modeling the term placenta without adjusting for potential maternal/fetal physiological variables; nevertheless it has enhanced our understanding of human placental transfer of key substances, including immune complexes in the context of maternal disease (May et al., 2009; Hutson et al., 2011; Wilcox et al., 2017). Further studies following maternal immunization would be timely.

Recently, there have been collaborative efforts (e.g., The Human Placenta Project) to optimize our existing models and/or apply novel technologies to more accurately recapitulate the diverse cell types and complex interactions at the materno-fetal interface. Key examples include co-cultures cultivating both trophoblastic and endothelial cells on a membrane; three-dimensional trophoblast organoids; computational modeling of tissue dynamics and blood flow; and bio-engineered tissue constructs (Guttmacher et al., 2014; Huckle, 2017; Aengenheister et al., 2018; Turco et al., 2018).

## Beyond Biology

With the considerable international interest in vaccination in pregnancy by academic researchers, industry and community stakeholders alike, it has become apparent that in addition to the multitude of biological challenges summarized above, considerable implementation challenges also need to be addressed (Bonhoeffer et al., 2016; Munoz, 2018; Munoz et al., 2018). These relate to safety, equity of access, acceptability, prioritization, standardization of clinical diagnoses, use of not-yet licensed vaccines in emergency situations like Ebola or Lassa, to name but a few. There is a need for pregnancy registers to capture long-term outcomes, beyond clinical trials. How to set this up in LMICs where, ultimately, these vaccines might have the largest impact on maternal and neonatal health, but where the background rates of pregnancy and neonatal outcomes are not as reliably captured as in HICs, remains a considerable challenge. Many other reviews have comprehensively addressed these topics.

## Joining Forces to Address Both Biological and Implementation Challenges

Over the last 2 years, IMPRINT, the international IMmunising PRegnant Women and INfant NeTwork has brought together investigators and stakeholders in 50 countries across the globe to conduct research into specific biological and implementation challenges. The Pregnancy Research Ethics for Vaccines, Epidemics, and New Technologies (PREVENT) team has set out a framework for ethical consideration of inclusion of pregnant women in clinical trials of new vaccines (Prevent, 2020); more specific roadmaps for the introduction of vaccines against GBS (WHO GBS vaccine research development technical roadmap, 2020) and RSV have also been developed (PATH, 2018). The Bill and Melinda Gates Foundation (BMGF) and WHO have each committed significant resources to the subject and supported the academic community, with industry as a further key stakeholder, in driving forward the agenda to develop and implement safe and effective vaccines in pregnancy.

**FIGURE 1:**
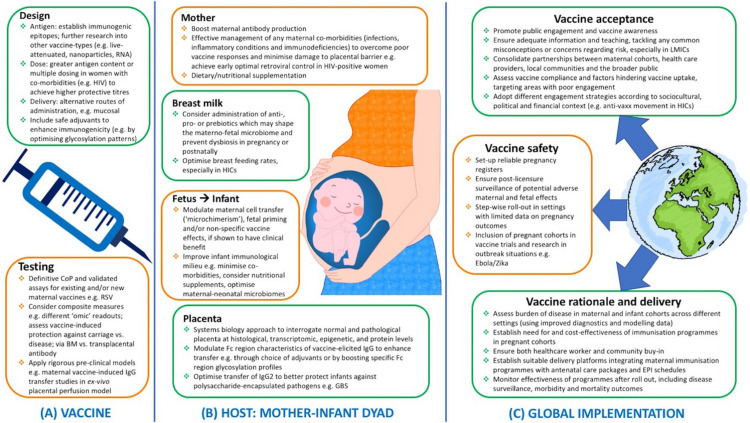
Potential future strategies to ensure effective maternal immunization programs worldwide, from **(A)** vaccine design and testing, **(B)** optimal host responses at the maternal/fetal/infant levels, and **(C)** successful implementation at the global scale with consideration of key financial, political, socio-cultural, logistical influencing factors. LMICs, low- and middle-income countries; CoP, correlate of protection; BM, breast milk; EPI, expanded program of immunization; GBS, Group B streptococcus; HIC, high-income countries; HIV, human immunodeficiency virus; Ig, immunoglobulin; RSV, respiratory syncytial virus.

Figure 1 summarizes potential future strategies to optimize the success of maternal immunization programs worldwide, addressing both biological (vaccine and host-related) factors and implementation challenges.

## Conclusion

In Boxes 1, 2 below, we have outlined key avenues of research, either targeted to the materno-fetal interface or the infant, which could enhance our understanding of the underlying immunobiology and inform design and testing of the next generation of safe and effective vaccines in pregnancy.

As we have summarized in this review, significant progress has been achieved to understand how mother and child are immunologically linked, thereby optimizing vaccine design to effectively and safely target this materno/fetal dyad. Much more remains to be done in this exciting area of vaccinology, which has already achieved unprecedented and laudable multi-stakeholder engagement.

## Author Contributions

BK developed the concept for the review and provided senior support. AS conducted the literature review and wrote the first draft. Both authors contributed to writing the manuscript and reviewed the final draft.

## Conflict of Interest

BK’s institution receives funding from a variety of donors, including industry for the conduct of vaccine-related research in women and children. The remaining author declares that the research was conducted in the absence of any commercial or financial relationships that could be construed as a potential conflict of interest.
